# Large language model-based paper classification framework with key-insight extraction and confidence-weighted voting

**DOI:** 10.1017/rsm.2026.10094

**Published:** 2026-04-22

**Authors:** Zihan Song, Shan Huang, Ngeemasara Thapa, Xin Zhang, Byung-Kwon Park, Jie Lu, Wenyang Li, Wenbin Liu, Bei Zhan, Jianfei Li

**Affiliations:** 1 College of Industrial Internet, Hubei Engineering Institute, China; 2 Research and Development, Center for Emergency Medicine Advancement, Nepal; 3 Department of Management Information Systems, Dong-A University, Republic of Korea; 4 Department of External Cooperation, Zhejiang Academy of Industrial Internet Development, China

**Keywords:** artificial intelligence, evidence synthesis, large language model, literature screening, paper classification, systematic review

## Abstract

Systematic reviews (SRs) are critical for evidence-based research but are time-consuming and labor-intensive. The rapid expansion of academic publications further challenges the performance and applicability of existing screening and classification methods. While large language models (LLMs) present new opportunities for automation, limited research has examined whether they can achieve classification performance comparable to human reviewers in large-scale, multi-class settings. With the goal of improving classification performance, we proposed an LLM-based framework that leverages full-text key-insight extraction to enhance literature classification. We constructed a manually curated dataset of 900 articles from 17 published SRs to quantitatively evaluate the classification capabilities of LLMs. The results provided empirical evidence of LLMs’ potential in supporting large-scale SRs and introduced a practical pathway for improving efficiency and reliability in evidence synthesis. Empirical results showed that key-insight-based classification (KBC) significantly outperforms abstract-based classification (ABC). We implemented a confidence-weighted voting (CWV) mechanism using multiple LLMs to improve robustness. The CWV method achieved the highest macro *F*1-score of 0.796, substantially exceeding KBC (0.732), ABC (0.676), and unsupervised K-means clustering (0.446). By employing zero-shot LLMs, our approach demonstrated the potential for enhanced adaptability across diverse domains and classification tasks without requiring fine-tuning, demonstrating that a carefully designed pipeline can enable LLMs to achieve classification performance comparable to human reviewers.

## Highlights

### What is already known

- Traditional systematic review screening is labor-intensive, and existing machine learning methods are largely restricted to abstract-level data and depend on domain-specific corpora.

### What is new

- A novel LLM framework utilizing full-text “key-insight” extraction instead of simple abstract analysis.
- Performance significantly improved from a 0.676 macro *F*1-score (abstract-based method) to 0.796 (confidence-weighted voting method).

### Potential impact for *RSM* readers

- Reduces dependency on manual labeling and specialized models in evidence synthesis.
- Enables high-precision, cross-disciplinary paper classification without task-specific fine-tuning.

## 1 Introduction

A systematic review (SR) is an academic research methodology that systematically and objectively identifies, evaluates, and synthesizes all available research evidence pertinent to a particular topic or question.^1^ ^–^ ^3^ SRs play an essential role in supporting evidence-based decision-making. However, it is time- and resource-intensive, typically requiring 6–18 months to complete from protocol initiation to submission for publication.^4^ One of the most crucial steps is title and abstract screening, where researchers manually assess potentially eligible studies retrieved through structured keyword searches.^5^ On average, screening 60–120 records require approximately 1 hour.^5^ ^–^ ^7^ However, as the volume of papers increases, the risk of human error increases, driven by reviewer fatigue and cognitive overload.^8^

To reduce workload while maintaining decision performance, researchers have explored the use of text mining and machine learning (ML) techniques.^9^ ^,^ ^10^ Notable examples include Abstrackr, which can reduce screening effort by up to 67%,^11^ and Ryaan, which achieves a 20–54% reduction.^12^ Despite these advances, traditional supervised ML approaches are often domain-specific and dependent on labeled datasets. While unsupervised methods like k-means reveal latent structures without labeled data, their interpretability and parameter sensitivity constrain their utility as substitutes for manual screening.^13^

Recent advances in large language models (LLMs) enable efficient, accurate evidence synthesis by performing zero-shot reasoning, semantic understanding, and extracting structured data from unstructured text—reducing the need for manual annotation and task-specific labels.^14^ These capabilities provide a basis for developing end-to-end automated SR pipelines that integrate literature screening, information extraction, and classification—potentially minimizing the requirement for manual annotation.

At present, most of the research have employed LLMs for screening processes of title and abstract. For instance, Gargari et al.^15^ used GPT-3.5 for title and abstract screening achieving a sensitivity of 69%, whereas another GPT-3.5 study reported a higher sensitivity (97.1%) but low specificity (37.7%).^16^ Similarly, GPT-4 has also been employed as a tool for title and abstract screening, reporting varying sensitivities, 42–50%^17^ and 76%.^18^ Overall, these studies illustrate substantial promise but also reveal considerable variability, with specificity values in some cases below 40%. A key limitation is the reliance on title and abstract information alone, which restricts an LLM’s ability to fully capture study context, methodology, and findings—factors critical for accurate inclusion decisions. Although full-text–based classification could theoretically yield superior performance, its feasibility is constrained by LLM’s context length limitations and associated computational costs.

In our previous work,^19^ we demonstrated the feasibility of LLMs to extract key insights—including research questions, methodologies, and findings—from full-text articles, achieving 97% agreement with human experts (relevance score). This suggests that a key-insight-driven workflow offers a viable alternative to sole reliance on titles and abstracts. Building on this foundation, the present study aims to explore the potential of LLMs in managing complex academic paper classification tasks, specifically addressing the semantic limitations of traditional automated methods. We propose a novel classification paradigm that transitions from surface-level metadata analysis to deep-text semantic synthesis. To this end, this study pursues the following objectives:

1. To develop a classification protocol centered on full-text “key-insight” extraction, utilizing LLMs to automatically distill research objectives, methodologies, and core findings. This overcomes the information bottleneck inherent in abstract-only classification and enables a more granular characterization of scientific literature.
2. To implement a novel confidence-weighted voting (CWV) mechanism that mitigates the stochastic instability of individual LLMs in multi-class settings. By aggregating decisions from multiple state-of-the-art models and assigning weights based on empirical validation performance, we establish a robust decision-making framework.
3. To establish a rigorous quantitative benchmark against expert-level “gold standards.” Using manually curated classification schemes from published SRs, we evaluate the framework’s performance in large-scale, real-world academic contexts to provide empirical evidence for the viability of LLM-based end-to-end automation.

## 2 Related work

In automated SR, paper classification constitutes a core component of evidence synthesis, directly influencing the performance of downstream tasks such as information extraction and trend analysis. Existing approaches to automated classification can be broadly categorized into three developmental stages: (i) rule-based and traditional ML methods, (ii) deep learning and pre-trained language models (PLMs), and (iii) emerging LLM methods. Table 1 outlines representative studies, including dataset size, features, classification labels, best-performing techniques, and results.

**Table 1:** Overview of methods for automated paper classification in automated SR Table 1 Long description.

Work	Sample size	Features	Classification labels	Best method	Results
Mustafa et al.^20^	86,166	Title, abstract, keywords, general terms	11 top-level ACM categories	K-Nearest neighbor	*F*1-score: 0.88
Tzika-Kostopoulou et al.^41^	2,671	Title, abstract, keywords,	8 transportation research topics	Naïve Bayes	Accuracy: 0.91
Kandimalla et al.^21^	~45,000,000	Abstract	104 subject categories	Deep attentive neural networks	*F*1-score: 0.76
Lu et al.^22^	4,000	Title, abstract, keywords, WoS categories	Binary classification	Ensemble model (SciBERT + decision tree + keyword matching + support vector machine)	*F*1-score: 0.94
Raja et al.^42^	1,000	Title, abstract	19 categories summarized by experts	Bidirectional and auto-regressive transformers	*F*1-score: 0.85
Rao et al.^43^	788	Title, abstract, keywords	A total of 44 hierarchical classes	Fine-tuned recurrent neural network	Accuracy: 0.36
López-Pineda et al.^24^	984	Title, abstract	Binary classification	Lamma 3	Sensitivity: 0.77 Specificity: 0.91
Ali et al.^44^	31,175	Title, abstract	Binary classification	Active learning	WSS@95%:0.75
Dai et al.^45^	113	Title, abstract, full text	Binary classification	GPT–4o with revised chain-of-thought prompt	AUC: 1.00
Colangelo et al.^46^	33,057	Title, abstract	Binary classification	Claude 3 Haiku	*F*1-score: 0.85–1.0

Early approaches predominantly relied on rule-based systems and conventional ML, using keyword matching, statistical feature extraction, and supervised learning. For example, Mustafa et al.^20^ applied K-Nearest Neighbor to the ACM dataset with features from titles, abstracts, keywords, and common terms, achieving an *F*1-score of 0.88. Such methods depend heavily on metadata, which is often inconsistent or incomplete and have limited semantic understanding, making cross-domain classification difficult.

The second phase of development adopted deep learning and PLMs (e.g., BERT and its variants), enabling more effective semantic capture for classification and relevance prediction. Kandimalla et al.^21^ employed a deep attention neural network to classify 45 million paper abstracts into 104 topics, achieving an *F*1-score of 0.76. Another study^22^ combined SciBERT, decision trees, keyword matching, and support vector machine for a binary artificial intelligence (AI)-related classification, reaching 0.94 *F*1-score. These methods capture semantics more effectively but require substantial labeled data and have limited cross-domain generalization.

The emergence of LLMs has led to a third paradigm shift in SR automation. Contemporary approaches leverage LLMs’ emergent capabilities in zero-shot learning and semantic reasoning to address longstanding challenges. Our previous work^19^ demonstrated that GPT-4 achieves 97% relevance score in extracting key insights from scientific articles through structured prompting. In clinical research, similar promising results were observed in trial eligibility screening using hybrid human–AI workflows, achieving a prevalence- and bias-adjusted k of 0.96 against consensus-based human decisions.^18^ Innovations in multimodal systems like Gemini-Pro,^23^ which integrates text and citation graph analysis, have reduced manual screening time compared to traditional methods. However, methods employed by LLM like LLaMA3, which achieved 77.5% sensitivity and 91.4% specificity using only titles and abstracts, often remain limited to surface-level features lacking deeper semantic and methodological insights from full texts.^24^ This limitation constrains both classification performance and interpretability. Frameworks such as TnT-LLM^25^ operationalize these capabilities through a phased architecture. In systematic review applications, their two-stage pipeline first generates domain-specific ontologies using GPT-4’s multi-step reasoning (achieving 87% taxonomy coverage) and then trains lightweight classifiers achieving 0.92 *F*1-score with 94% cost reduction versus pure-LLM classification. These advancements align with clinical SR requirements where dynamic label systems must adapt to evolving evidence hierarchies.

Despite these advancements, critical limitations remain. Most implementations concentrate on document-level processing rather than cross-document knowledge synthesis.^19^ This sequential, paper-by-paper workflows overlook the higher order relations among studies.^26^ Furthermore, existing evaluation frameworks tend to prioritize operational metrics (e.g., time savings) over epistemological soundness. This gap is particularly evident in complex review contexts where meaningful synthesis arises from the aggregation of insights across a large corpus. These limitations motivated our proposed system, which integrates key-insight extraction with classification to automatically generate literature structures akin to those produced by domain experts.

## 3 Methods

### 3.1 Overview

**Figure 1:**
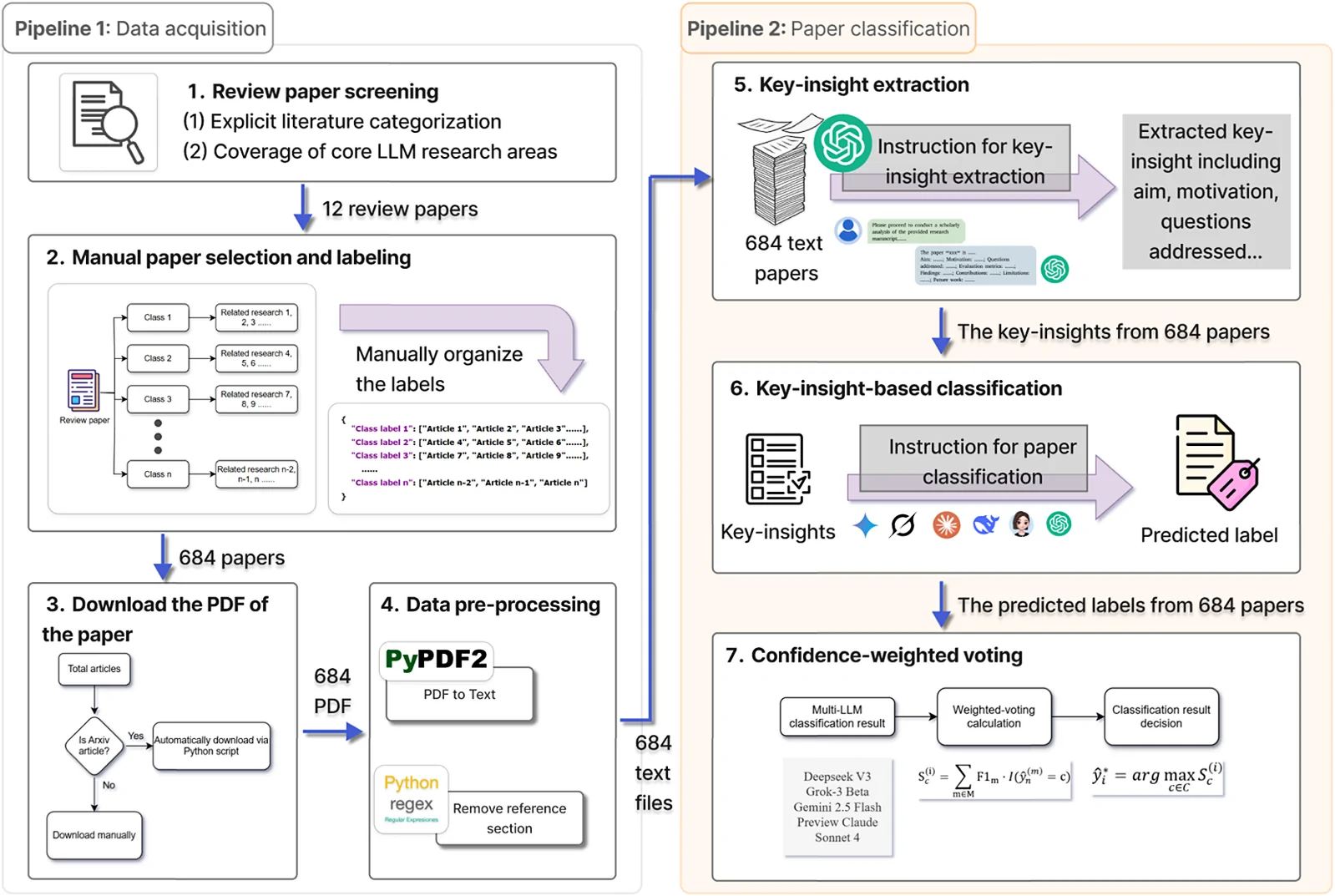
System architecture. Figure 1 Long description.

This study addressed the prevailing challenges of inefficiency and limited automation in conducting SRs by introducing a novel framework that leverages LLMs to integrate automated paper classification with key-insight extraction. To operate this framework, we constructed a two-stage pipeline. The pipeline combines expert-annotated labels with automated LLM-based processing. As illustrated in Figure 1, the overall workflow was divided into two main pipelines: data acquisition and paper classification. The left part of the figure details the manual and automated steps for assembling a labeled dataset. The right part visualizes the process of extracting key insights and performing classification using LLMs. Specifically, let $D={\left\{\left(x_{i},y_{i}\right)\right\}}_{i=1}^{N}$ denote the labeled dataset, where N denotes the total number of papers, $x_{i}$ represents the textual content of the i th article, and $y_{i}\in C$ is its expert-assigned category label, with C being the set of all possible categories.

Our proposed method introduced a two-step mapping process. First, we defined a key-insight extraction function g:x↦k , where *k* is a structured representation of the paper’s academic key insights—such as research objectives, question addressed, and findings—systematically summarized from the full text. Second, based on these extracted key insights, we employed a classification function $f^{\prime }:k\mapsto y$ to predict the category label y . Accordingly, the automated classification is formalized as the composite mapping:

(1) $$\begin{array}{r} f(x):=f^{\prime }\left(g(x)\right). \end{array}$$

The two-stage pipeline proceeded as follows: (i) extraction of expert-provided classification labels from review articles (where the cited literature is manually categorized in a structured JSON format; see Figure 1); (ii) automation of key-insight extraction from each paper using LLMs; and (iii) utilization of extracted key-insight input by final LLM-based classification, producing predictions that aimed to maximize the agreement with human expert labels, as quantified by the following metric:

(2) $$\begin{array}{r} \mathrm{Performance}=\frac{1}{N}\sum_{i=1}^{N}I\left(f^{\prime }\left(g\left(x_{i}\right)\right)=y_{i}\right), \end{array}$$

where I(⋅) is the indicator function.

### 3.2 Dataset

#### 3.2.1 Data acquisition

Obtaining labeled datasets is challenging in automated SRs. Traditional manual annotation methods require domain experts to spend substantial time screening and classifying literature. Meanwhile, unsupervised learning may produce clustering outcomes that are misaligned with the actual needs of academic fields.^27^ To address this issue, we utilized classification schemes extracted from existing literature review papers as ground-truth labels for evaluating algorithm performance.

Let $S={\left\{s_{j}\right\}}_{j=1}^{K}$ denote the set of selected survey papers, where K denotes the total number of survey papers, each providing a manually constructed classification scheme $C_{j}={\left\{c_{k}^{\left(j\right)}\right\}}_{k=1}^{n_{j}}$ . Each paper $s_{j}$ further specified a set of cited publications and their assigned categories as pairs $R_{j}={\left\{\left(r_{i}^{\left(j\right)},c_{k(i)}^{\left(j\right)}\right)\right\}}_{i=1}^{m_{j}}$ , where $r_{i}^{\left(j\right)}$ is a cited work in survey j , $c_{k\left(i\right)}^{\left(j\right)}$ its assigned category, $m_{j}$ denotes the number of primary research papers that are both cited and explicitly categorized in the j th survey paper $s_{j}$ . By aggregating all surveys, we obtain a labeled dataset:

(3) $$\begin{array}{r} D={⋃}_{j=1}^{K}R_{j}={\left\{\left(x_{n},y_{n}\right)\right\}}_{n=1}^{N}, \end{array}$$

where $x_{n}$ is the full text of the n -th cited paper, $y_{n}$ its category label, and N denotes the total number of papers.

*Survey paper selection criteria*: We identified relevant survey papers through Google Scholar using the search queries “LLM review” and “large language model literature review,” restricting the publication period to 2021–2025 and limiting the results to papers published in English. Survey papers were defined as structured surveys that provide explicit literature categorization, rather than strictly systematic reviews. The selected surveys met the following criteria: (i) Explicit literature categorization: Papers must provide structured classifications with clearly labeled citation-category mappings. (ii) Coverage of core LLM research areas: Surveys should focus on key technical aspects, applications, or challenges in LLMs. This process yielded 17 eligible survey papers and 900 cited research papers for dataset construction. Notably, we did not deliberately seek systematic reviews that flowing PRISMA standard. Among the 17 survey papers, only 2 qualify as systematic reviews with PRISMA-like rigor, reflecting the nascent stage of LLM literature.

*Data collection*: We extracted author-assigned category labels from the surveys, representing generalized classifications of literature within specific domains. We described all 17 review papers and their corresponding dataset information in Supplementary Table 1. For instance, Kaddour et al.^28^ defined 11 distinct categories—Chatbot, Computational Biology, Computer Programming, Creative Work, Knowledge Work, Law, Medicine, Reasoning, Robotics, Social Science, and Synthetic Training Data—each linked to relevant citations. Similarly, we organized these citations and their corresponding categories into our labeled dataset (Figure 1). These author-assigned categories were subsequently used as the ground truth to evaluate the performance of our multi-class classification. For each $\left(x_{n},y_{n}\right)$ in our dataset, the full-text PDF of $x_{n}$ was collected either via (1) automated retrieval using the Python ArXiv library^29^ or (2) manual retrieval for non-ArXiv sources. This dual sourcing ensured comprehensive coverage of all cited works.

#### 3.2.2 Data preprocessing

To facilitate automated key-insight extraction and ensure data quality, multiple preprocessing steps were applied to the collected dataset. To define the dataset for analysis, let $X={\left\{x_{n}^{\mathrm{raw}}\right\}}_{n=1}^{N}$ denote the set of text documents, where $x_{n}^{\mathrm{raw}}$ represents the full raw text extracted from the PDF [( $x_{n}^{\mathrm{raw}}=f_{\mathrm{PDF}}\left(x_{n}\right)]$ . Full raw text content was extracted from the full-text PDFs using the PyPDF2 library.^30^

To improve insight extraction efficiency and reduce irrelevant content, cleaning function $g_{clean}$ was applied to each document, removing reference section:

(4) $$\begin{array}{r} x_{n}^{\prime }=g_{\mathrm{clean}}\left(x_{n}^{\mathrm{raw}}\right),1\leq n\leq N, \end{array}$$

where $x_{n}^{\prime }$ is the filtered main body text.

Regular expressions were used for this cleaning step, substantially shortening the average length of article text strings from 76,941.6 to 59,144.3 characters, thereby removing redundant content and improving both the performance and efficiency of subsequent key-insight extraction.^19^

The final curated dataset was defined as $D_{\mathrm{final}}={\left\{\left(x_{n}^{\prime },y_{n}\right)\right\}}_{n=1}^{N}$ , derived from 17 survey papers that collectively defined 107 unique categories (averaging 8.3 per survey), with each category containing on average 7.5 articles. Out of the 900 research papers collected, approximately 46.3% was sourced from ArXiv.

### 3.3 Key-insight extraction

Key-insight extraction was the process of identifying valuable information contained in scientific articles,^31^ ^,^ ^32^ including research questions, methodologies, and stated objectives. From the SR perspective, key insights reflected focal areas of interest to researchers, such as organizing literature reviews based on research methodologies or research questions.

Mathematically, the key-insight extraction process can be formulated as a mapping $g_{\text{θ}}:X^{\prime }\to K$ , where $X^{\prime }={\left\{x_{n}^{\prime }\right\}}_{n=1}^{N}$ is the preprocessed full-text corpus and K denotes the space of key-insight representations. For each article, $k_{n}=g_{\theta }\left(x_{n}^{\prime }\right)$ is a structured sequence of key insights summarizing that article, that is,

(5) $$\begin{array}{r} k_{n}=\left\{k_{n}^{\left(1\right)},k_{n}^{\left(2\right)},\ldots ,k_{n}^{\left(n\right)}\right\}, \end{array}$$

where each $k_{n}^{\left(i\right)}$ corresponds to a specific insight, encompassing the aim, motivation, methodology, experimental results, contributions, and limitations.

Previous studies that employed manual comparison reported up to 97% similarity between the key insights extracted by GPT-4o and human experts from scientific articles.^19^ Accordingly, in this study, we utilized GPT-4o to extract key insights from all the selected articles, as detailed in Supplementary Table 2.

### 3.4 Key-insight-based classification

Classifying research papers remains challenging due to ambiguous category boundaries and overlapping research themes. Many papers span multiple topics, such as combining model development with application studies, making single-label assignment difficult. Additionally, variations in writing style and terminology increase the complexity of identifying key themes. Prior methods relied on titles, abstracts, or keywords, which may have overlooked critical methodological or conceptual details. As a result, classification based solely on limited textual cues can be shallow and inaccurate.

To address these limitations, we proposed a key-insight-based classification approach and hypothesized that it offers advantages over traditional methods by utilizing structured representations of each paper’s core contributions. Unlike conventional abstracts that often overlook methodological details, limitations, or application contexts, key insights provided a more comprehensive and structured summary of each paper. By explicitly capturing elements such as research objectives and findings, they offered a richer semantic foundation that may enhance model interpretability and classification performance.

Formally, this step implemented a two-stage mapping. For each preprocessed article $x_{n}^{\prime }$ , its key-insight representation $k_{n}=g_{\theta }\left(x_{n}^{\prime }\right)$ was first extracted as outlined in Section 3.3. Classification was then performed by an LLM-based classifier, which can be defined as a function:

(6) $$\begin{array}{r} {\hat{y}}_{n}=f_{\Phi }^{\prime }\left(k_{n}\right)=\arg\max_{c\in C}\mathrm{Pr}\left(c|k_{n},\Phi \right), \end{array}$$

where Φ represents the LLM’s parameters and prompt instructions, *c* is a candidate category, and C denotes the set of all possible categories. This approach used $k_{n}$ , a structured key insight extracted in the previous step, as the classification basis, rather than shallow cues such as the title or abstract.

To perform this classification, we employed six state-of-the-art LLMs as shown in Table 2. Each of these LLMs possessed distinct characteristics and capabilities that could affect classification performance. Model invocations were uniformly processed by OpenRouter,^33^ with the temperature parameter set to 0.7 and top-*p* set to 0.95 for each model to attain optimal performance through a balanced approach to predictability and creativity.^34^ A lower temperature value constrains the model’s randomness and creativity, whereas a higher temperature may result in excessively unstable outputs.

**Table 2:** LLMs used in this study Table 2 Long description.

Model name	Abbreviation	Company	Capacity	Max context length
claude-sonnet–4	Claude	Anthropic	Text generation, reasoning	200k
deepseek-v3–250324	Deepseek	Deepseek, Inc	Text generation	128k
doubao–1.5-pro–32k–250115	Doubao	ByteDance	Text generation	128k
gemini–2.5-flash-preview–05–20	Gemini	Google	Text generation, reasoning, multi-modal input	1024k
gpt–4o	GPT	OpenAI	Text generation, multi-modal input	128k
grok–3-beta	Grok	X-AI	Text generation, reasoning, multi-modal input	1024k

Specific instructions given to each model are described in Supplementary Table 3. The instruction’s design was optimized for robust classification through a two-fold strategy: the “System instruction” established a consistent interpretive framework by defining the LLM’s role as a meticulous literature researcher with strong thematic analysis capabilities, while the “User instruction” provided precise, contextualized input (title, key insight, category candidates, and descriptions) and mandated a structured JSON output. This methodological precision aimed to mitigate LLM interpretation variability and standardize response formats for subsequent analysis.

### 3.5 Confidence-weighted voting

To further enhance the robustness and trustworthiness of paper classification, we introduced a confidence-weighted voting framework informed by validation set performance. Instead of treating each model’s output as equally reliable, we incorporated evidence-based weighting that reflects the actual performance of each language model for our specific task.

Let $M=m_{1},m_{2}\ldots ,m_{p}$ denote the set of selected LLMs and let $V={\left\{\left(x_{v},y_{v}\right)\right\}}_{v=1}^{V}$ denote a small, labeled validation set representative of the classification task. Each model m in M is tasked with predicting labels for V . We then calculated the classification performance (macro *F*1-score) of each model and used this performance as the model’s overall reliability score. To avoid degradation in ensemble quality, due to poorly performing models, we referenced Section 4.2’s comparative experiments and excluded LLMs with significantly lower macro *F*1-scores on the validation set. Specifically, only models with macro *F*1-scores above a practical threshold were retained in the voting ensemble; this led us to select GPT, Grok, Gemini, and Claude. Deepseek and Doubao, which had notably lower macro *F*1-scores under our evaluation, were omitted from the final voting pool.

During the classification stage, each LLM was assigned a predicted category ${\hat{y}}_{n}^{\left(m\right)}$ for each article $x_{i}^{\prime }$ . Rather than simple majority voting, we assigned each model’s vote a weight, specifically its validation macro *F*1-score, $F1_{m}$ . The aggregated score for assigning article $x_{i}^{\prime }$ to category c∈C was defined as follows:

(7) $$\begin{array}{r} S_{c}^{\left(i\right)}=\sum_{m\in M}\mathrm{Macro}\;F1-{\mathrm{score}}_{m}\cdot I\left({\hat{y}}_{n}^{\left(m\right)}=c\right), \end{array}$$

where ${\hat{y}}_{n}^{\left(m\right)}$ denotes the predicted category assigned to $x_{n}^{\prime }$ and $S_{c}^{\left(i\right)}$ represents the aggregated weighted score for assigning class *c* to the *i*th article.

The final category assignment for $x_{i}^{\prime }$ is then determined as follows:

(8) $$\begin{array}{r} {\hat{y}}_{i}^{*}=\arg\max_{c\in C}S_{c}^{\left(i\right)}. \end{array}$$

This strategy ensured that predictions from more accurate models exerted proportionally greater influence on the outcome while still preserving the benefits of model diversity. In case of tie between categories after weighted voting, the output of the single model with the highest $S_{c}^{\left(i\right)}$ was chosen as the tiebreaker.

## 4 Performance evaluation

### 4.1 Evaluation metrics

In this study, the paper classification task was framed as a multi-class classification problem. During the performance evaluation phase, our primary focus was to assess whether the proposed system produced results like those provided by the authors of the review papers. To this end, we used the accuracy, macro precision, macro recall, and macro *F*1-score as the evaluation metric. The definition of these metrics are as follows:

For each class i in a multi-class classification task, we compute its precision $P_{i}$ , recall $R_{i}$ , and F1 score $F1_{i}$ :

(9) $$\begin{array}{r} P_{i}=\frac{{\mathrm{TP}}_{i}}{{\mathrm{TP}}_{i}+{\mathrm{FP}}_{i}}, \end{array}$$

(10) $$\begin{array}{r} R_{i}=\frac{{\mathrm{TP}}_{i}}{{\mathrm{TP}}_{i}+{\mathrm{FN}}_{i}}, \end{array}$$

(11) $$\begin{array}{r} F1_{i}=2\times \frac{P_{i}\times R_{i}}{P_{i}+R_{i}}, \end{array}$$

where TP refers to true positives, FP to false positives, and FN to false negatives. The macro precision, macro recall score, and macro *F*1-score are then defined as follows:

(12) $$\begin{array}{r} \mathrm{Macro}\;\mathrm{precision}=\frac{1}{n}\sum_{i=1}^{n}P_{i}, \end{array}$$

(13) $$\begin{array}{r} \mathrm{Macro}\;\mathrm{recall}=\frac{1}{n}\sum_{i=1}^{n}R_{i}, \end{array}$$

(14) $$\begin{array}{r} \mathrm{Macro}\;F1-\mathrm{score}=\frac{1}{n}\sum_{i=1}^{n}F1_{i}, \end{array}$$

where n denotes the total number of classes.

### 4.2 Confidence estimation on small-sample evaluation

To ensure the scientific rigor and effectiveness of the confidence-weighted voting mechanism, we conducted a detailed evaluation of several mainstream LLMs on a small-scale validation set, with a particular focus on employing the macro *F*1-score as the confidence weight. We used the key insight from 57 randomly selected papers as independent classification inputs to compare the multi-class performance of six prominent LLMs. To prevent data leakage, the test dataset used in the results, as presented in Section 4.3, does not include these 57 papers.

Evaluation metrics included accuracy, macro precision, macro recall, and macro *F*1-score. The detailed results are presented in Table 3. The assessment results revealed discernible differences in macro *F*1-scores among the various models. The GPT model achieved the best performance with a macro *F*1-score of 0.848. Grok, Gemini, and Claude also performed robustly, each yielding macro *F*1-scores close to or above 0.8. In contrast, Deepseek and Doubao exhibited lower macro *F*1-scores, registering 0.787 and 0.738, respectively.

**Table 3:** Classification performance of different LLMs on the small-scale validation set Table 3 Long description.

Model	Accuracy	Precision	Recall	*F*1-score
GPT	0.877	0.891	0.846	0.848
Grok	0.860	0.895	0.830	0.817
Gemini	0.842	0.838	0.816	0.800
Claude	0.842	0.880	0.799	0.799
Deepseek	0.842	0.868	0.792	0.787
Doubao	0.807	0.857	0.739	0.738

*Note:* The above accuracy values reflect overall accuracy. Precision, Recall, and *F*1-score are computed as macro average metrics.

To improve the overall robustness and quality of the weighted voting mechanism, we used each model’s macro *F*1-score on the small validation set as its confidence score. The ensemble then assigned weights to each model based on these confidence scores. Due to considerably inferior macro *F*1-scores compared to the other models, Deepseek and Doubao were excluded from the final weighted voting, aiming to prevent degradation of the ensemble’s performance. Only the classification outputs from GPT, Grok, Gemini, and Claude were retained for the final ensemble.

### 4.3 Comparative analysis of classification approaches

#### 4.3.1 Overview of comparative methods

In recent years, natural language processing (NLP) tasks have increasingly favored zero-shot approaches that do not require parameter training or fine-tuning, to enhance generalizability and convenience. Traditional methods^20^ ^–^ ^22^ required model training before deployment and were less adaptable to new domains or classification schemes. Accordingly, all comparative methods in this study—including abstract-based LLM classification, key-insight-based LLM classification, and unsupervised K-means clustering—were implemented without additional training or fine-tuning, thus providing a realistic evaluation of the proposed system’s applicability in current automated systematic reviews.

To evaluate the performance of the proposed system, we introduced several comparative methods as benchmarks:

- *K-means clustering (KMC)*: K-means is a widely adopted unsupervised clustering algorithm,^35^ commonly used in literature clustering tasks. The performance of this method served as a baseline for the classification task and was used to evaluate the performance of other methods. The paper abstracts were encoded using Salesforce’s SFR-Embedding-2_R model, followed by dimensionality reduction using principal component analysis,^36^ retaining 90% of the variance. Clustering was performed separately for each review’s dataset using scikit-learn library, with *k* set to the number of categories in that review (e.g., *k* = 11 for Review 1), and other parameters at defaults. As KMC does not yield predefined class labels, we employed the Hungarian matching algorithm^37^ to align the resulting clusters with the ground truth labels based on maximum overlap. KMC provides a conventional unsupervised baseline, emphasizing the value of our zero-shot LLM approaches over purely data-driven clustering without expert labels.
- *Abstract-based classification (ABC)*: This method utilized LLMs to classify each paper based solely on its title and abstract, as presented in Supplementary Table 4.
- *Key-insight-based classification (KBC)*: In this approach, the LLM performed classification based on extracted key insights from each paper, as presented in Supplementary Table 3. Compared to the abstract-based classification, this approach enabled a more systematic examination of the potential advantages conferred by using key insights in place of original abstracts.
- *Confidence-weighted voting method (CWV)*: The method proposed in Section 3.5.

#### 4.3.2 Results

Table 4 represents the results for the four approaches. For ABC, accuracy ranged from 0.688 (Grok) to 0.726 (Claude), with macro *F*1-scores between 0. 654 and 0. 704, respectively. The average macro *F*1-score was 0.676. This indicates that titles and abstracts alone offer limited detail for accurate classification. With KBC, accuracies increased to range of 0.734–0.768, and macro *F*1-scores improved to between 0.708 and 0.749. The richer context from key insights improved classification quality. KMC achieved 0.524 accuracy and 0.446 macro *F*1-score, indicating that unsupervised clustering here fails to capture category boundaries effectively. The CWV method reached 0.797 accuracy, 0.805 precision, 0.809 recall, and a 0.796 macro *F*1-score. Weighted ensembles of strong LLMs delivered the best overall performance.

**Table 4:** Performance of paper classification methods Table 4 Long description.

Methods	Models	Accuracy	Precision	Recall	*F*1-score
ABC	Deepseek	0.693	0.693	0.705	0.664
	Grok	0.688	0.678	0.697	0.654
	Gemini	0.702	0.722	0.716	0.683
	Claude	0.726	0.73	0.736	0.704
	GPT	0.696	0.725	0.705	0.669
	Doubao	0.708	0.729	0.715	0.679
	Average	0.702	0.713	0.712	0.676
KBC	Deepseek	0.734	0.736	0.755	0.708
	Grok	0.766	0.766	0.79	0.745
	Gemini	0.752	0.754	0.773	0.734
	Claude	0.768	0.762	0.783	0.749
	GPT	0.749	0.757	0.774	0.732
	Doubao	0.759	0.759	0.771	0.73
	Average	0.755	0.756	0.774	0.732
KMC		0.524	0.475	0.484	0.446
CWV		**0.797**	**0.805**	**0.809**	**0.796**

*Note:* The above accuracy values reflect overall accuracy. Precision, Recall, and *F*1-score are computed as macro average metrics. ABC: abstract-based classification; CWV: confidence-weighed voting method; KBC: key-insight-based classification; KMC: K-means clustering.

**Figure 2:**
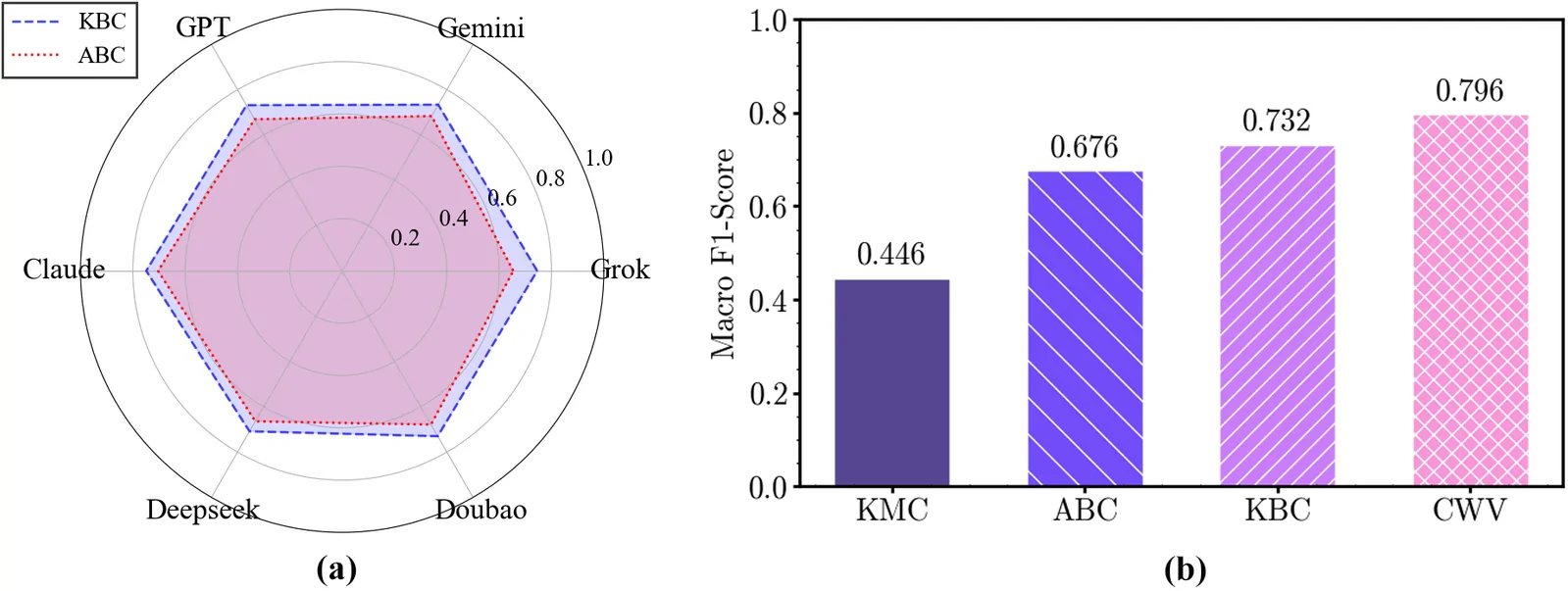
(a) Macro F1-score differences of candidate LLMs: key insight versus abstract classification. (b) Comparison of the macro F1-scores across the four methods. ABC: abstract-based classification; CWV: confidence-weighed voting method; KBC: key-insight-based classification; KMC: K-means clustering. Figure 2 Long description.

Figure 2 summarizes the overall performance trends from different approaches. As shown in Figure 2a, for all candidate LLMs, the KBC consistently outperformed the ABC, indicating that key insights provided a more informative basis for classification than full abstracts. Figure 2b shows that, in terms of the macro *F*1-score, CWV achieved the highest score (0.796), substantially surpassing both single-model classification approaches (KBC: 0.732; ABC: 0.676) and the unsupervised K-means clustering baseline (KMC: 0.446). These results demonstrated a clear and consistent improvement when combining multiple LLMs through confidence-weighted voting, highlighting the advantage of integrating complementary model strengths.

**Figure 3:**
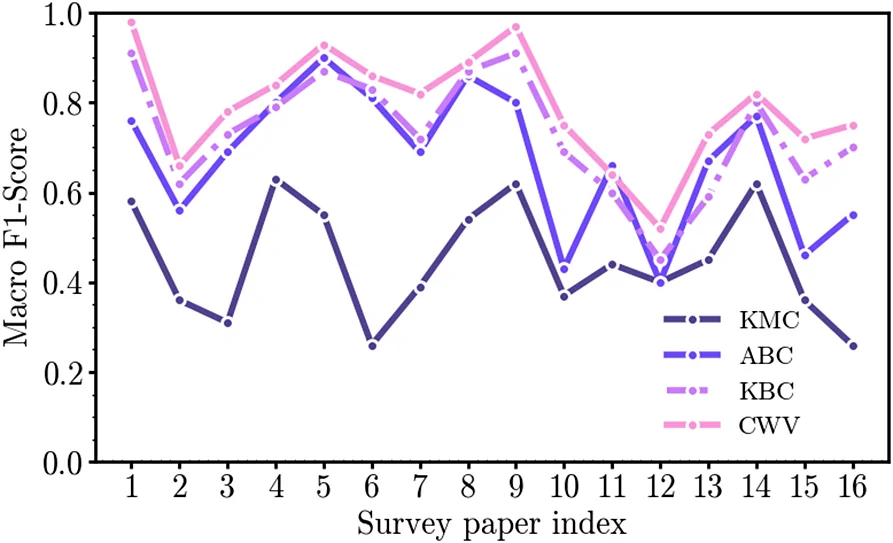
Average macro F1-scores of the KMC, ABC, KBC, and CWV methods on datasets corresponding to different survey papers, where ABC and KBC results represent averages across all models. ABC: abstract-based classification; CWV: confidence-weighed voting method; KBC: key-insight-based classification; KMC: K-means clustering. Figure 3 Long description.

Figure 3 illustrates the average macro *F*1-scores of the KMC, ABC, KBC, and CWV methods across datasets corresponding to 16 survey papers (where the results for ABC and KBC represent averages across all models). Overall, KMC exhibited significantly lower performance than LLM-based methods on all datasets, with substantial variability, indicating that purely unsupervised clustering struggles to stably capture the complex and fine-grained classification boundaries across different surveys. Compared to KMC, ABC showed marked improvements; however, it still suffers from performance deficiencies on multiple datasets (e.g., surveys 7, 10, and 15), underscoring the limitations of relying solely on the representational capacity of titles and abstracts. KBC outperformed ABC on most survey datasets (except surveys 6, 11, and 13), highlighting the beneficial role of key insights in cross-survey classification tasks. The CWV method performed comparably to KBC, slightly surpassing it on all datasets, which demonstrates the advantages of multi-agent voting approaches.

#### 4.3.3 Statistical significance of results

Given the proximity of the observed performance scores, it was essential to assess whether these differences were statistically meaningful. Due to the presence of natural boundaries in the data—potentially resulting in skewed distributions—we employed the Wilcoxon signed-rank test to evaluate differences across LLM outputs. This non-parametric test was selected because it does not assume normality, making it better suited for data with distributional irregularities. The null hypothesis posited no statistically significant differences in macro *F*1-score, while the alternative hypothesis suggested that such differences did exist. Supplementary Table 5 summarizes the test results across all evaluated methods. All pairwise comparisons yielded statistically significant differences (*p* < 0.05), indicating that each method differs meaningfully from the others. These results confirmed that subtle variations in performance were not due to random chance but a reflection of consistent methodological distinctions.

#### 4.3.4 Error overlap analysis across classification methods

**Figure 4:**
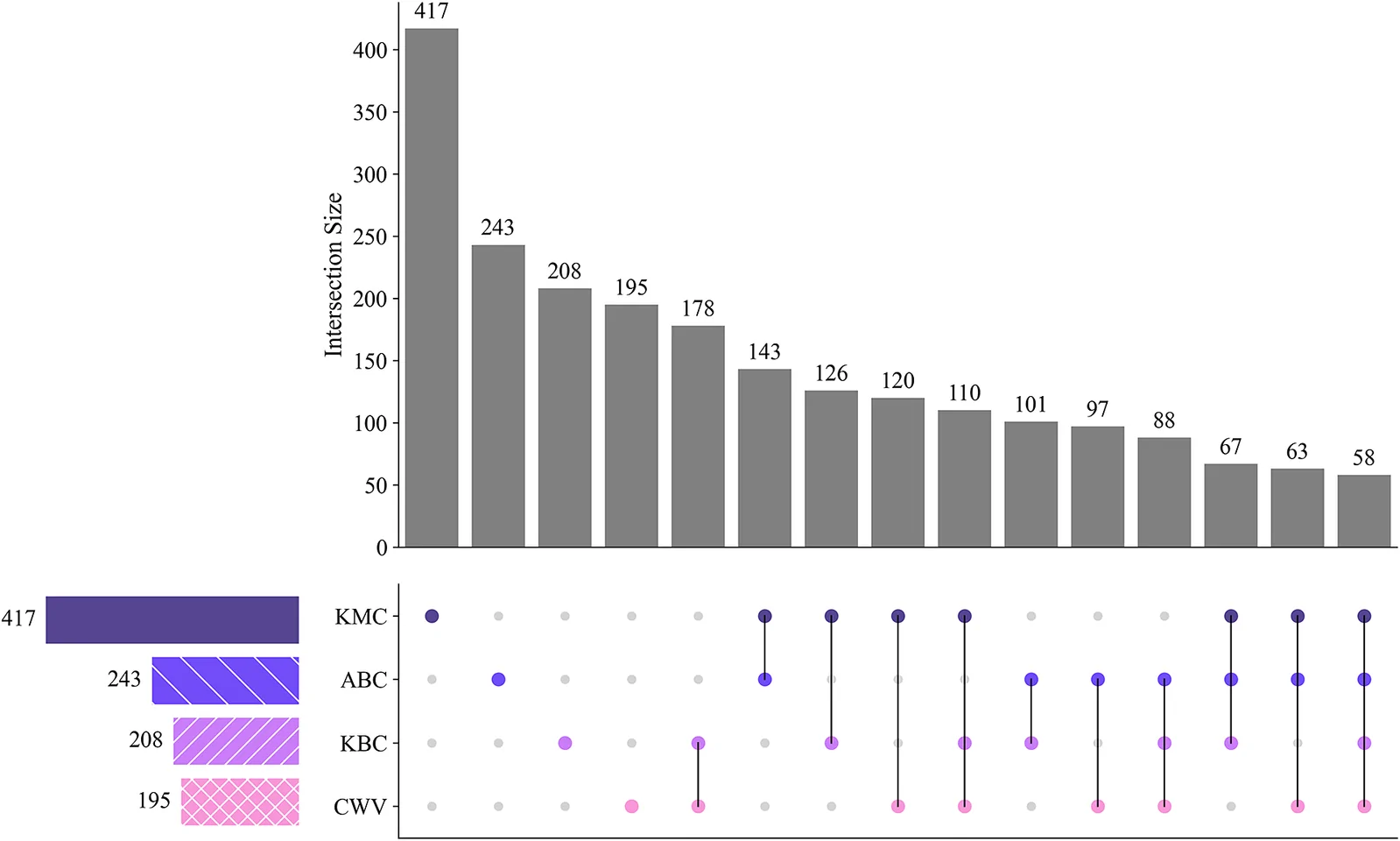
UpSet plot of misclassification overlaps among classification approaches. ABC: abstract-based classification; CWV: confidence-weighed voting method; KBC: key-insight-based classification; KMC: K-means clustering. Figure 4 Long description.

To further investigate the similarities and differences in error patterns among the classification approaches, we performed an intersection analysis of misclassified samples using an UpSet plot (Figure 4). For the LLM-based settings, Claude was selected as the representative model for the ABC and GPT for the KBC. The results revealed that the KMC method produced the largest number of unique misclassifications (417), with substantial overlap with the errors of other methods, indicating its limited ability to capture nuanced and complex category boundaries without supervision. Among the zero-shot LLM approaches, ABC yielded 243 uniquely misclassified cases, whereas KBC reduced this number to 208, reflecting the advantage of leveraging structured key insights over raw abstracts in providing richer contextual grounding for classification. In contrast, the proposed CWV approach further reduced misclassifications beyond both ABC and KBC, demonstrating its ability to integrate complementary strengths of multiple high-performing LLMs and mitigate errors that persisted in single-model settings.

Notably, the intersection analysis also revealed a core set of misclassified samples (58) common to all four methods. These papers frequently span multiple research themes or exhibit substantial content overlapping across categories, making them difficult to assign to a single class unambiguously. This finding highlighted an inherent limitation of single-label multi-class approaches when applied to cross-domain or thematically overlapping literature, suggesting that multi-class classification or category ambiguity modeling may be more appropriate in such cases.

## 5 Discussion

### 5.1 Overview of core findings

This study presents a novel LLM-based framework designed to automate the complex task of paper classification within SRs. Our approach primarily leveraged the generalization capabilities of LLMs. Compared with traditional language models, LLMs achieved significant advancements in both the performance and span of contextual understanding.^38^ These improvements made it feasible to perform deeper and more meaningful analyses of article content. Unlike prior research such as ChatPDF,^39^ which applied LLMs for content-based querying, or other emerging tools like Abstrackr^11^ that focused on predicting researchers’ interest in academic papers, our study leverages LLMs to generate reviewer-like outputs, facilitating expert-aligned evaluations in rigorous SRs.

Our empirical evaluation across a manually curated benchmark of 900 articles from 17 survey papers yielded several critical insights:

First, KBC outperforms ABC. As shown in Table 4, moving from metadata-level analysis to full-text synthesis improved the macro *F*1-score by 8.2% on average. In specialized domains like LLM research, abstracts frequently lack the granular methodological details required for precise categorization. By extracting and structuring core findings into key insights, the classifiers gain access to the high-density evidence needed to resolve category ambiguity.

Second, the CWV mechanism effectively mitigates the inherent instability of individual zero-shot LLMs. While single-model performance varied, the aggregated CWV approach achieved a macro *F*1-score of 0.796, approaching human-expert levels. The effectiveness of a static weighting scheme across 17 heterogeneous datasets suggests that the different reasoning patterns of various LLMs are complementary. This ensemble-based approach provides a reliable validation mechanism for SR workflows, where classification accuracy is critical for downstream synthesis.^40^

Finally, the comparison with the KMC baseline highlights a shift in automation paradigms. Despite using state-of-the-art semantic embeddings, unsupervised clustering correlates poorly with expert-defined taxonomies (macro *F*1: 0.446). This disparity underscores that scientific classification requires alignment with specific, objective-driven ontologies rather than just identifying statistical patterns. Our zero-shot framework addresses this by using pre-defined category definitions as semantic references, enabling it to adapt to diverse review goals without the need for manual labeling or domain-specific fine-tuning.

### 5.2 The superiority of key-insight extraction

The central finding of this study is the measurable performance advantage of KBC over ABC. This superiority is consistently observed across 13 out of 16 survey datasets (Figure 3), suggesting that the benefit of using distilled full-text data is systemic rather than dataset-specific.

The performance gap between ABC and KBC (averaging an 8.2% improvement in macro *F*1-score) can be analyzed through the lens of information sufficiency. While abstracts are designed to provide a high-level summary, they are often subject to length constraints imposed by journals (typically 200–400 words), leading to the omission of specific methodological parameters, secondary findings, or tool-specific implementation details. In our dataset, the average input string length for ABC was 3879.5 ± 1260.3 characters, whereas KBC provided 5867.8 ± 1284.8 characters. However, this gain is not merely a product of increased token count. Abstracts are typically highly compressed due to strict word limits, which often forces the omission of critical technical implementations, such as specific algorithmic variants or detailed benchmark results. The KBC mechanism overcomes this inherent information scarcity by retrieving and reconstructing research objectives, technical methodologies, specific metrics, and core findings from the full-text body. This process does not simply reduce text; rather, it introduces high-value details that are absent from metadata and reorganizes them into a structured format, significantly increasing the density of discriminative information.

This structural refinement is particularly effective when navigating categories with high semantic overlap. In Survey-1 (Supplementary Table 1), which focuses on Parameter-Efficient Fine-Tuning (PEFT), numerous papers are described at the abstract level using generic terms like “optimizing large-scale models,” making it difficult for ABC to distinguish between specific technical pathways. We provide a concrete example in Supplementary Table 6, comparing the abstract and key insights of the paper “Raise a Child in Large Language Model: Towards Effective and Generalizable Fine-tuning.” In this instance, while the abstract refers broadly to “strategically masking” a subset of parameters, the key insights successfully retrieve crucial details from the full text, such as the two distinct variants: the task-free Child-TuningF (utilizing a Bernoulli distribution) and the task-driven Child-TuningD (utilizing Fisher Information). Crucially, the key insights also capture the method’s divergence from model pruning—noting that it retains the full network during inference—and its orthogonality to existing techniques.

These specific features retrieved from the full-text body provide the LLM with unambiguous evidence for categorization. By transforming implicit features into explicit structural insights, the KBC framework significantly enhances the model’s ability to differentiate between fine-grained technological categories that appear indistinguishable in a metadata-only analysis. Furthermore, the structured evidence layer provided by KBC enhances the transparency of the automated classification process. In an SR workflow, researchers can directly audit the logical consistency between the extracted “key insights” and the final categorical assignment. By transforming implicit full-text features into explicit, structured insights, the KBC framework not only compensates for the poverty of information in abstracts but also provides an auditable chain of evidence, making automated decision-making more robust and aligned with the rigorous requirements of scientific evidence synthesis.

### 5.3 Collective decision logic and deployment in systematic reviews

This study integrated predictions from multiple LLMs through CWV, significantly enhancing classification stability. Although a static weighting strategy based on validation set *F*1 scores was employed, the performance gains remain substantial. This improvement stems primarily from the complementary bias of different models when processing complex semantics. Due to divergences in pre-training corpora and reasoning logic, individual models often exhibit specific inferential biases when handling interdisciplinary or semantically overlapping literature categories. The CWV mechanism effectively leverages the consensus of high-performance models, such as GPT-4o and Claude 3.5, mitigating classification errors caused by stochasticity or context-window limitations of a single model through weighted allocation. In practical deployment, the application of this ensemble mode must balance precision against computational cost; thus, we propose the CWV framework as an extensible solution that can be dynamically regulated based on task stages and user requirements.

For individual researchers or small teams, the primary value of CWV lies in providing a low-cost, automated “cross-check” mechanism, aimed at mitigating systematic misjudgments by a single model that are often difficult to detect due to limited human resources. In such lightweight scenarios, users are not required to maintain multi-model operations throughout the entire workflow; instead, a phased strategy can be implemented. During the initial screening stage of a SR, when faced with thousands of retrieved records, a low-cost ABC paired with a single model can be utilized to maximize recall while controlling inference overhead. Only upon transition to the full-text analysis stage—where the sample size has been pruned to hundreds and requires high-precision adjudication—is the multi-model decision-making process, based on key-insight extraction, activated. This on-demand invocation logic ensures that individual researchers can achieve expert-level reliability and consistency with controllable API expenditures.

For large-scale academic institutions or commercial developers, the engineering significance of this framework is manifested in its optimization of classification stability and throughput for complex literature streams. In commercial deployments, the “semantic compression layer” facilitated by key-insight extraction can structure raw PDFs—which often contain tens of thousands of characters—into compact representations of approximately 5,000 characters. This directly reduces the total token consumption of concurrent multi-model adjudications and bypasses the computational bottlenecks associated with long-context processing. Furthermore, developers of large-scale systems can monitor the consensus level of voting distributions to identify disciplinary fields with low classification consistency, thereby routing controversial samples arising from LLM uncertainty to human experts for manual review. This modular design, tailored for late-stage precision classification and label alignment, allows researchers to switch flexibly between KBC and CWV methods based on the specific scale and budget of the SR. This ensures that the framework maintains both scientific rigor and engineering feasibility when processing massive volumes of academic literature.

### 5.4 Limitations and directions for future research

Despite demonstrating the efficacy of key-insight extraction and weighted voting mechanisms, this study is subject to several limitations. First, regarding dataset construction, although we expanded the expert-annotated set to include 900 papers, approximately 46.3% of the data originated from the ArXiv platform. While this distribution reflects the rapid iteration of research in the LLM field, ArXiv abstracts often exhibit a concise style characteristic of specific disciplines (e.g., computer science and mathematics). This may differ from the more structured and formal abstracts typical of biomedical or social science journals. This limitation in data sources suggests that future research should apply the framework to disciplines with traditions of longer, more complex abstracts to evaluate its generalizability across diverse writing norms. Second, this study is predicated on the classification criteria of previously published survey papers; consequently, the upper bound of our findings is constrained by the inter-annotator agreement of the original authors. Human multi-class classification inherently involves significant subjective variance. The stabilization of macro *F*1 scores for literature classification between 0.7 and 0.8 largely reflects the blurred boundaries caused by interdisciplinary overlaps—a challenge that cannot be eliminated through technical optimizations alone. On the other hand, although the proposed system showed strong alignment with human-assigned categories, it currently assumes a static and mutually exclusive set of classification labels. Future research could explore hierarchical or multi-label classification frameworks to accommodate the complex and overlapping nature of scholarly work.

In light of these challenges, future research should shift from static evaluation toward more interactive and dynamic systems. The current framework assumes a predefined and static classification labeling system; however, in actual SR workflows, taxonomies often evolve as evidence accumulates. Future work could explore the introduction of an ontology-driven closed-loop mechanism, allowing LLMs to automatically propose new categories or restructure classification hierarchies when a significant portion of the literature fails to align with existing labels. Furthermore, to address resource-balancing pressures in large-scale applications, the development of adaptive model-selection logic holds substantial engineering value. Specifically, the system could dynamically determine whether to invoke high-cost, high-tier models or multi-model voting based on initial confidence scores. This would enable an efficiency optimization whereby “low-uncertainty samples follow a single-model pipeline, while highly controversial samples trigger collective decision logic.” Finally, establishing a human-in-the-loop evaluation standard that incorporates interaction between human experts and LLMs will be critical. Researching how LLM-generated key insights can assist researchers in more rapidly correcting classification biases will provide a key pathway for transitioning automated systematic reviews from mere tool-assisted tasks to a paradigm of human–AI collaboration.

## 6 Conclusion

This study presented a novel framework for automated paper classification in SRs using LLMs to extract key insights from full-text articles. The proposed CWV method approach significantly outperformed traditional methods, achieving a macro *F*1-score of 0.796 compared to 0.676 for ABS. CWV mechanism successfully integrated multiple LLMs, reducing classification errors and enhancing robustness. Statistical validation confirmed these performance improvements, demonstrating that key insights provide superior classification foundations than surface-level features. Although computational costs and annotation subjectivity still remain as challenges, this research provides a promising foundation for more efficient automated systematic review systems. Future work should explore hierarchical classification frameworks to enhance scalability across diverse academic domains.

## Supporting information

10.1017/rsm.2026.10094.sm001 Song et al. supplementary material Song et al. supplementary material

## Data Availability

The data that support the findings of this study are openly available at https://doi.org/10.5281/zenodo.19221294.

## Supplementary material

To view supplementary material for this article, please visit http://doi.org/10.1017/rsm.2026.10094.

## Long descriptions

### Table 1 Long description

The table consists of six columns: Work, Sample size, Features, Classification labels, Best method, and Results.

* Mustafa et al. used a sample of 86,166 with title, abstract, keywords, and general terms to classify 11 top-level A C M categories using K-Nearest neighbor, achieving an F 1-score of 0.88.

* Tzika-Kostopoulou et al. used 2,671 samples with title, abstract, and keywords for 8 transportation research topics using Naive Bayes, achieving an accuracy of 0.91.

* Kandimalla et al. used approximately 45,000,000 abstracts for 104 subject categories using deep attentive neural networks, achieving an F 1-score of 0.76.

* Lu et al. used 4,000 samples with title, abstract, keywords, and W o S categories for binary classification using an ensemble model (Sci B E R T plus decision tree plus keyword matching plus support vector machine), achieving an F 1-score of 0.94.

* Raja et al. used 1,000 samples with title and abstract for 19 categories using bidirectional and auto-regressive transformers, achieving an F 1-score of 0.85.

* Rao et al. used 788 samples with title, abstract, and keywords for 44 hierarchical classes using a fine-tuned recurrent neural network, achieving an accuracy of 0.36.

* Lopez-Pineda et al. used 984 samples with title and abstract for binary classification using Lamma 3, achieving sensitivity of 0.77 and specificity of 0.91.

* Ali et al. used 31,175 samples with title and abstract for binary classification using active learning, achieving a W S S at 95 percent of 0.75.

* Dai et al. used 113 samples with title, abstract, and full text for binary classification using G P T-4o with revised chain-of-thought prompt, achieving an A U C of 1.00.

* Colangelo et al. used 33,057 samples with title and abstract for binary classification using Claude 3 Haiku, achieving an F 1-score between 0.85 and 1.0.

Navigate back to Table 1.

### Figure 1 Long description

The flowchart is divided into two main sections.

Pipeline 1: Data acquisition includes steps 1 through 4.

Step 1. Review paper screening: Focuses on explicit literature categorization and coverage of core L L M research areas, resulting in 12 review papers.

Step 2. Manual paper selection and labeling: A diagram shows a review paper branching into classes 1 through n, which are then manually organized into a J S O N-style list of articles. This results in 684 papers.

Step 3. Download the P D F of the paper: A decision tree checks if an article is from Arxiv to determine if it is downloaded via Python script or manually.

Step 4. Data pre-processing: Uses Py P D F 2 for P D F to text conversion and Python regex to remove reference sections, resulting in 684 text files.

Pipeline 2: Paper classification includes steps 5 through 7.

Step 5. Key-insight extraction: 684 text papers are processed through an instruction for key-insight extraction using a generative A I model to extract aims, motivations, and questions.

Step 6. Key-insight-based classification: Extracted insights are passed through a classification instruction to generate a predicted label.

Step 7. Confidence-weighted voting: Multi-L L M classification results from Deepseek V 3, Grok-3 Beta, Gemini 2.5 Flash, Preview Claude, and Sonnet 4 undergo a weighted-voting calculation. The final classification result decision is determined by the formula y-hat sub i equals arg max over c in C of S sub c super open parenthesis i close parenthesis, where S sub c super open parenthesis i close parenthesis equals the summation over m in M of F 1 sub m times I open parenthesis y-hat sub n super open parenthesis m close parenthesis equals c close parenthesis.

Navigate back to Figure 1.

### Table 2 Long description

The table consists of five columns and six data rows.

* Row 1: Model name claude-sonnet-4, Abbreviation Claude, Company Anthropic, Capacity Text generation and reasoning, Max context length 200k.

* Row 2: Model name deepseek-v3-250324, Abbreviation Deepseek, Company Deepseek, Inc, Capacity Text generation, Max context length 128k.

* Row 3: Model name doubao-1.5-pro-32k-250115, Abbreviation Doubao, Company ByteDance, Capacity Text generation, Max context length 128k.

* Row 4: Model name gemini-2.5-flash-preview-05-20, Abbreviation Gemini, Company Google, Capacity Text generation, reasoning, and multi-modal input, Max context length 1024k.

* Row 5: Model name g p t-4o, Abbreviation G P T, Company OpenAI, Capacity Text generation and multi-modal input, Max context length 128k.

* Row 6: Model name grok-3-beta, Abbreviation Grok, Company X-A I, Capacity Text generation, reasoning, and multi-modal input, Max context length 1024k.

Navigate back to Table 2.

### Table 3 Long description

The table consists of five columns: Model, Accuracy, Precision, Recall, and F 1-score. The data is organized into six rows representing different Large Language Models:

* G P T: Accuracy 0.877, Precision 0.891, Recall 0.846, F 1-score 0.848.

* Grok: Accuracy 0.860, Precision 0.895, Recall 0.830, F 1-score 0.817.

* Gemini: Accuracy 0.842, Precision 0.838, Recall 0.816, F 1-score 0.800.

* Claude: Accuracy 0.842, Precision 0.880, Recall 0.799, F 1-score 0.799.

* Deepseek: Accuracy 0.842, Precision 0.868, Recall 0.792, F 1-score 0.787.

* Doubao: Accuracy 0.807, Precision 0.857, Recall 0.739, F 1-score 0.738.

A note specifies that Accuracy reflects overall accuracy, while Precision, Recall, and F 1-score are computed as macro average metrics.

Navigate back to Table 3.

### Table 4 Long description

The table presents performance metrics for four paper classification methods.

1. A B C (Abstract-Based Classification) evaluated across six models:

- Deepseek: Accuracy 0.693, Precision 0.693, Recall 0.705, F 1 score 0.664.

- Grok: Accuracy 0.688, Precision 0.678, Recall 0.697, F 1 score 0.654.

- Gemini: Accuracy 0.702, Precision 0.722, Recall 0.716, F 1 score 0.683.

- Claude: Accuracy 0.726, Precision 0.73, Recall 0.736, F 1 score 0.704.

- GPT: Accuracy 0.696, Precision 0.725, Recall 0.705, F 1 score 0.669.

- Doubao: Accuracy 0.708, Precision 0.729, Recall 0.715, F 1 score 0.679.

- Average: Accuracy 0.702, Precision 0.713, Recall 0.712, F 1 score 0.676.

2. K B C (Key-Insight-Based Classification) evaluated across six models:

- Deepseek: Accuracy 0.734, Precision 0.736, Recall 0.755, F 1 score 0.708.

- Grok: Accuracy 0.766, Precision 0.766, Recall 0.79, F 1 score 0.745.

- Gemini: Accuracy 0.752, Precision 0.754, Recall 0.773, F 1 score 0.734.

- Claude: Accuracy 0.768, Precision 0.762, Recall 0.783, F 1 score 0.749.

- GPT: Accuracy 0.749, Precision 0.757, Recall 0.774, F 1 score 0.732.

- Doubao: Accuracy 0.759, Precision 0.759, Recall 0.771, F 1 score 0.730.

- Average: Accuracy 0.755, Precision 0.756, Recall 0.774, F 1 score 0.732.

3. K M C (K-means clustering):

- Accuracy 0.524, Precision 0.475, Recall 0.484, F 1 score 0.446.

4. C W V (Confidence-Weighed Voting):

- Accuracy 0.797, Precision 0.805, Recall 0.809, F 1 score 0.796.

C W V shows the highest performance across all metrics, while K M C shows the lowest.

Navigate back to Table 4.

### Figure 2 Long description

Panel a is a radar chart with six axes representing L L M s: G P T, Gemini, Grok, Doubao, Deepseek, and Claude. The radial scale ranges from 0.0 at the center to 1.0 at the perimeter in increments of 0.2. Two shaded hexagonal regions are plotted. The inner region, bounded by a red dotted line, represents A B C. The outer region, bounded by a blue dashed line, represents K B C. The K B C area consistently exceeds the A B C area across all six models, indicating higher scores.

Panel b is a bar chart with the y-axis labeled Macro F 1-Score from 0.0 to 1.0. Four bars are arranged from left to right:

* K M C: solid dark purple bar with a value of 0.446.

* A B C: blue bar with diagonal stripes with a value of 0.676.

* K B C: light purple bar with diagonal stripes with a value of 0.732.

* C W V: pink bar with a cross-hatch pattern with a value of 0.796.

The trend shows a steady increase in performance from K M C to C W V.

Navigate back to Figure 2.

### Figure 3 Long description

The vertical Y axis is labeled Macro F 1 Score ranging from 0.0 to 1.0. The horizontal X axis is labeled Survey paper index with integer values from 1 to 16. Four distinct lines represent the methods.

* K M C (dark purple dashed line with dots) consistently shows the lowest performance, fluctuating between approximately 0.25 and 0.65. It peaks at index 4 and 14.

* A B C (solid blue line with dots) generally tracks above K M C, ranging from 0.4 to 0.9. It shows significant dips at indices 2, 10, 12, and 15.

* K B C (light purple dash-dot line with dots) closely follows the A B C trend but often maintains slightly higher scores, particularly between indices 1 and 9.

* C W V (solid pink line with dots) represents the highest performing method across almost all indices, frequently exceeding 0.8 and reaching near 1.0 at indices 1 and 9.

All four methods show a synchronized sharp decline at index 12 before recovering at index 14.

Navigate back to Figure 3.

### Figure 4 Long description

The plot is divided into three main sections.

1. Top Section: A vertical bar chart with the Y-axis labeled Intersection Size ranging from 0 to 400. There are 15 gray bars representing the size of specific overlaps. From left to right, the values are 417, 243, 208, 195, 178, 143, 126, 120, 110, 101, 97, 88, 67, 63, and 58.

2. Bottom-Left Section: Four horizontal bars representing the total set size for each classification approach.

- K M C: 417 (solid dark purple)

- A B C: 243 (diagonal striped purple)

- K B C: 208 (diagonal striped light purple)

- C W V: 195 (cross-hatched pink)

3. Bottom-Right Section: A matrix of dots aligned with the vertical bars above. Darker colored dots indicate the sets included in that specific intersection, while light gray dots indicate exclusion. Vertical lines connect the colored dots to show multi-set overlaps.

- The first four bars represent unique misclassifications for K M C, A B C, K B C, and C W V respectively.

- The fifth bar (178) shows an overlap between K B C and C W V.

- The sixth bar (143) shows an overlap between K M C and A B C.

- Subsequent bars show increasingly complex overlaps, with the final bar (58) representing an intersection of all four methods (K M C, A B C, K B C, and C W V).

Navigate back to Figure 4.
