# Supplementary material for: Large language model-based paper classification framework with key-insight extraction and confidence-weighted voting
Source: Res Synth Methods. 2026 Apr 22;17(5):999–1017. doi: 10.1017/rsm.2026.10094 (PMC13732363; doi:10.1017/rsm.2026.10094)
Supplement: Supplementary file 1 [file S1759287926100945sup001.pdf]

Supplementary Tables

Table 1. Dataset information

Survey paper index	DOI	Dataset size	Category list
1	https://doi.org/10.48550/arXiv.2312.12148	57	AdapterBasedFineTuning, BiasUpdate, DeltaWeightMasking, HybridFT_AutomaticCombination, HybridFT_ManualCombination, LoRA Derivatives, LowRankDecomposition, OtherAdditiveFT, PretrainedWeightMasking, SoftPromptBasedFT, UnifiedFineTuning
2	https://doi.org/10.48550/arXiv.2307.10169	96	Chatbot, Computational Biology, Computer programming, Creative work, Knowledge work, law, Medicine, Reasoning, Robotics, SocialScience, Synthetic Training Data
3	https://doi.org/10.1007/s11704-024-40231-1	49	CS & SE, Documentation and data management Experiment Assistant, Industrial automation, Jurisprudence, Natural Science education, Political Science and Economy, Psychology, Research Assistant, Robotics & embodied ai, Social Simulation

4	https://doi.org/10.1016/j.eswa.2021.114820	34	Explainability and Trustworthiness, Knowledge Representation, Learning and Inference, Logic and Reasoning
5	https://doi.org/10.48550/arXiv.2501.05435	19	Aggregations, Modifications to DL Architecture, Pipelines, Rule-Constrained Model
6	10.1109/MIPRO60963.2024.10569741	28	Explainability, KnowledgeProcessing, LogicalReasoning, NeuralAutomata, ProbabilisticReasoning
7	https://doi.org/10.48550/arXiv.2411.04383	70	Argumentation, MathematicalReasoning, ProgrammingSystems, Question-Answering, Robotics&Control, ScientificDiscovery, Vision-Language Analysis&Reasoning, VisualSceneUnderstanding
8	10.1109/TNNLS.2024.3420218	43	LogicalConstraints, LogicallyInformedEmbedding, RuleLearning
9	10.1109/TAI.2024.3351798	21	Constraint Solving, Over-Approximation, Over-Approximation & Constraint Solving, Search-Based, Search-Based & Constraint Solving

10	https://doi.org/10.1145/3719664	44	On-device applications, Pre-deployment techniques, Runtime optimization
11	https://doi.org/10.1145/3641289	91	Benchmarks and Datasets, Evaluation of Natural Language Processing Tasks, Multimodal and Tool Application Evaluation, Resoning capacity evaluation, Robustness, Ethics, and Bias Assessment
12	https://doi.org/10.1038/s44222-025-00279-5	117	Basic Model Architecture and Principles, Clinical application scenarios, Development Methods of Medical Large Language Models, Ethics, Safety and Regulations, Evaluation Benchmarks and Metrics
13	https://doi.org/10.3390/app15148103	105	Agriculture, Art, Business, Education, Energy, Finance, Industry, Law, Media, Medicine, Programming, Research, Tourism
14	10.1109/ACCESS.2025.3573955	52	Machine Translation, Text Generation, Text Classification, Text Summarization, Sentiment Analysis, Spam Filtering, Question Answering, Specialized domains such as healthcare, education
15	10.1109/ACCESS.2024.3365742	21	Agriculture, Bio-Medical and Healthcare, Business, Education, Social Media

16	https://doi.org/10.1145/3777411	24	Human-Crafted, Synthetic Data（Distillation）, Synthetic Data（Self-Improvement)）
17	https://doi.org/10.1145/3712001	27	Backdoor Attack, Data Poisoning Attack, Jailbreaking Attack, Prompt Injection

Table 2. Instructions for key-insight extraction.

System instruction	You are a meticulous literature researcher. You possess strong thematic analysis skills, enabling you to accurately extract key information and core points.
User instruction	Start with the file name in quotes, followed by a short overview paragraph (2–4 sentences) describing the paper's topic, main contribution, and approach. After the overview, add the heading ### Key Points:. Present the key insights as a numbered list. Each numbered item should have: A bolded title describing the section (e.g., Motivation and Background, Methodology, Experimental Results, Ablation Studies and Analysis, Limitations, Conclusion). Several bullet points or sentences elaborating on that aspect in your own words (avoid direct quotations from the paper). Cover at least the following aspects: Motivation and Background: Why the research was done, context, prior work, and the problem addressed. Methodology: How the proposed method works and what makes it unique. Experimental Results: Key benchmarks, datasets, model performance, and comparisons. Ablation Studies and Analysis:

	Additional experiments, observations, and explanations. Limitations: Weaknesses, assumptions, or untested scenarios. Conclusion: Final takeaways, contributions, and potential future work.<br>Keep the writing concise, factual, and clear. Summarize complex ideas in plain English without omitting crucial technical details.<br>Research manuscript: [Research manuscript]

Table 3. Instructions for paper classification based on key-insight.

System instruction	You are a meticulous literature researcher. You possess strong thematic analysis capabilities and can determine the category of a paper based on its abstract. Your responsibility is to classify papers according to the key-insight and title provided by users.
User instruction	Please determine the appropriate category for this article based on its title and key-insight.<br>Below are the category candidates: [Insert category candidates]<br>Below are the descriptions of each category: [Insert category description]<br>Article title: [Insert article title]<br>Article key-insight: [Insert key-insight]<br>Return your answer in JSON format. Here is an example of JSON format:<br>{<br>"Choice": "Your choice",

	"Reason": "The reason you made your choice"<br>}

Table 4. Instructions for paper classification based on abstract.

System instruction	You are a meticulous literature researcher. You possess strong thematic analysis capabilities and can determine the category of a paper based on its abstract. Your responsibility is to classify papers according to the title and abstract provided by users.
User instruction	Please determine the appropriate category for this article based on its abstract.<br>Below are the category candidates: [Insert category candidates]<br>Below are the descriptions of each category: [Insert category description]<br>Article title: [Insert article title]<br>Article abstract: [Insert article abstract]<br>Return your answer in JSON format. Here is an example of JSON format:<br>{<br>"Choice": "Your choice",<br>"Reason": "The reason you made your choice"<br>}

Table 5. Results of the Wilcoxon Signed-Rank Test.

	KMC	ABS	KBS	CWV
KMC	-	0.001	0.001	0.001
ABS	-	-	0.029	0.025
KBS	-	-	-	0.025
CWV	-	-	-	-

Table 6. Comparison between the Abstract and key-insights in the paper "Raise a Child in Large Language Model: Towards Effective and Generalizable Fine-tuning" (10.18653/v1/2021.emnlp-main.749)

Abstract	Recent pretrained language models extend from millions to billions of parameters. Thus the need to fine-tune an extremely large pretrained model with a limited training corpus arises in various downstream tasks. In this paper, we propose a straightforward yet effective fine-tuning technique, Child-Tuning, which updates a subset of parameters (called child network) of large pretrained models via strategically masking out the gradients of the non-child network during the backward process. Experiments on various downstream tasks in GLUE benchmark show that Child-Tuning consistently outperforms the vanilla fine-tuning by 1.5 8.6 average score among four different pretrained models, and surpasses the prior fine-tuning techniques by 0.6 1.3 points. Furthermore, empirical results on domain transfer and task transfer show that Child-Tuning can obtain better generalization performance by large margins.
Key-insights	The research article "Raise a Child in Large Language Model: Towards Effective and Generalizable Fine-Tuning" by Runxin Xu et al. introduces a novel fine-tuning technique called Child-Tuning for large pretrained language models (PLMs). The technique addresses the challenges of fine-tuning models with millions to billions of parameters, especially when training data is limited, which often leads to overfitting and poor generalization.<br>Key-insights:<br>1. Problem Statement: Fine-tuning large PLMs with limited data can lead to overfitting and poor generalization; conventional methods often update all parameters, which is unnecessary and can degrade performance.

2. Child-Tuning Technique: Child-Tuning updates only a subset of parameters (the "child network") by masking out the gradients of the non-child network during backpropagation. It includes two variants: Child-TuningF (a task-free approach using a Bernoulli distribution) and Child-TuningD (a task-driven approach using Fisher Information).
3. Methodology: The technique involves a gradient mask applied during backpropagation to update only the child network. The task-free variant introduces randomness to maintain gradient expectations, while the task-driven variant uses task-specific data to determine important parameters.
4. Experiments and Results: Conducted on the GLUE benchmark and additional NLI datasets, Child-Tuning consistently outperformed vanilla fine-tuning and other methods across various tasks and models like BERT, XLNet, RoBERTa, and ELECTRA. It demonstrated better generalization to out-of-domain data and across tasks.
5. Comparison with Prior Methods: Compared against methods like weight decay, top-k tuning, Mixout, RecAdam, and R3F, Child-Tuning (especially the task-driven variant) showed superior performance and can be integrated with other methods for further improvements.
6. Low-Resource Scenarios: Child-Tuning was effective in low-resource settings, reducing overfitting and improving performance compared to vanilla fine-tuning.
7. Discussion: Unlike model pruning, Child-Tuning retains the full network during inference. Ablation studies show the task-driven child network is crucial for performance, and overlapping analysis revealed higher child network overlap for similar tasks.
8. Conclusion: Child-Tuning is a simple yet effective technique for fine-tuning large PLMs, enhancing both performance and generalization. It is orthogonal to existing methods, allowing for integration to achieve further gains.

The study highlights the potential of Child-Tuning to improve the adaptability and robustness of large language models in various downstream applications, particularly when data is scarce.
